# Myocardial Recovery and Relapse in Heart Failure With Improved Ejection Fraction

**DOI:** 10.1007/s11936-024-01038-2

**Published:** 2024-05-20

**Authors:** Nandan Kodur, W. H. Wilson Tang

**Affiliations:** 1 Cleveland Clinic Lerner College of Medicine of Case Western Reserve University, 9500 Euclid Avenue, Desk J3-4, Cleveland, OH, US; 2 Department of Cardiovascular Medicine, Heart, Vascular and Thoracic Institute, Cleveland Clinic, Cleveland, OH, US

**Keywords:** Myocardial recovery, Myocardial relapse, Heart failure with improved ejection fraction, HFimpEF

## Abstract

**Purpose of review:**

The purpose of this review is to discuss myocardial recovery in heart failure with reduced ejection fraction (HFrEF) and to summarize the contemporary insights regarding heart failure with improved ejection fraction (HFimpEF).

**Recent findings:**

Improvement in left ventricular ejection fraction (LVEF ≥ 40%) with improved prognosis can be achieved in one out of three (10–40%) patients with HFrEF treated with guideline-directed medical therapy. Clinical predictors include non-ischemic etiology of HFrEF, less abnormal blood or imaging biomarkers, and lack of specific pathogenic genetic variants. However, a subset of patients may ultimately relapse, suggesting that many patients are merely in remission rather than having fully recovered.

**Summary:**

Patients with HFimpEF have improved prognosis but nonetheless remain at risk of relapse and long-term adverse events. Future studies will hopefully chart the natural history of HFimpEF and identify clinical predictors such as blood or novel imaging biomarkers that distinguish subgroups of patients based on differential trajectory and prognosis.

## Introduction

Heart failure (HF) is a common and debilitating condition that affects over 6.2 million Americans adults and 26 million adults worldwide [1, 2]. The classification of HF has traditionally been imaging-centric based on left ventricular ejection fraction (LVEF), with LVEF ≤ 40% being defined as HF with reduced ejection fraction (HFrEF), LVEF 41–49% as HF with mildly reduced ejection fraction (HFmrEF), and LVEF ≥ 50% as HF with preserved ejection fraction (HFpEF) [3]. Among these categories, HFrEF is currently most amenable to treatment, with disease-modifying, guideline-directed medical therapy (GDMT) for HFrEF entailing quadruple therapy consisting of a beta-blocker, renin-angiotensin (RAS) inhibitor, mineralocorticoid receptor antagonist (MRA), and sodium-glucose cotransporter-2 inhibitor (SGLT2i) [4]. There is growing recognition that a subset of patients with HFrEF who receive GDMT or device therapy experience improvement of LVEF to > 40%, a phenomenon that was initially termed HF with recovered ejection fraction (HFrecEF). This term, however, has recently been changed to HF with improved ejection fraction (HFimpEF) to underscore that, in many cases, mere remission rather than full recovery has occurred [3]. Specifically, HFimpEF is defined by the following three criteria: a baseline LVEF ≤ 40%, a ≥ 10% absolute improvement in LVEF, and a subsequent measurement of LVEF > 40% [3]. Given that HFimpEF appears to be independently associated with improved prognosis compared with persistent HFrEF, there has been increasing research seeking to characterize HFimpEF and its clinical predictors [5–9]. Despite increased characterization of HFimpEF, however, this clinical entity still remains poorly understood, which in turn has precluded optimal management of patients with this condition. In this review, we discuss the current state of knowledge regarding HFimpEF with respect to terminology and definitions, epidemiology, clinical predictors, physiologic mechanisms, and clinical course.

## Terminology and definitions for improvement of myocardial function

Even before broad adoption of GDMT, spontaneous improvement of LVEF with or without medical therapy had been observed in patients with HFrEF. A term for improvement of myocardial function in HFrEF, as assessed by LVEF, was first introduced in a 2011 retrospective cohort study by Punnoose et al., in which patients who previously had LVEF < 40% but subsequently achieved LVEF ≥ 40% were considered to have “HF with recovered ejection fraction” [10]. Almost a decade later, in 2020, a consensus term for improvement of myocardial function was established in a *JACC* Scientific Expert Panel, in which HFrecEF was defined by three criteria: 1) baseline LVEF ≤ 40%, 2) ≥ 10% absolute improvement in LVEF, and 3) a subsequent measurement of LVEF > 40% [11•]. Notably, the LVEF threshold of 40% was arbitrarily defined, presumably to align with the clinical indications for GDMT in HFrEF. During this time, there was also broader recognition that such improvements in LVEF might not be sustainable and that a subset of patients may experience deterioration of LVEF again with or without de-escalation of GDMT. In 2021, a writing committee consisting of members from several major HF societies published a consensus document on universal definitions and classification of HF in which it was proposed that the term HFrecEF be replaced with the term HFimpEF in order to better convey that patients who experience resolution of HF signs and symptoms as well as normalization of myocardial structure and function are nonetheless still at considerable risk of relapse [3, 12••]. In this vein, the authors also stress the importance of referring to HF with these clinical improvements as “HF in remission” as opposed to “recovered HF”, with this latter term only being used for select patients whose HFrEF was induced by a fully reversible cause (e.g., tachycardia-mediated cardiomyopathy, stress-induced/Takotsubo cardiomyopathy, or alcoholic cardiomyopathy) [3, 12••].

Notably, the current definition of HFimpEF does not differentiate between patients with subsequent LVEF > 40% versus LVEF ≥ 50% (with “normal” LVEF also being arbitrarily defined clinically, as what is considered a normal value for LVEF is largely population based and differs across various professional society guidelines), given that a subset of patients with complete normalization of LVEF to ≥ 50% along with normalization of left ventricular volumes may still be at considerable risk of relapse following discontinuation of GDMT [12••, 13•]. This highlights the limitations of using conventional imaging-derived measurements of cardiac structure and function to determine whether full myocardial recovery has occurred, and underscores the need for greater insights into biological signatures and novel biomarkers for assessing likelihood of sustained myocardial recovery.

## Historical context and epidemiology of HFimpEF

The prevalence of HFimpEF among patients diagnosed with HFrEF has been estimated to range between 10–40%, with the specific value depending on the research cohort, study design, and definition of HFimpEF used [5–9, 14]. Improvement of LVEF in patients with HFrEF has been described as early as 1985, in which 22 of 42 patients with congestive cardiomyopathy who were longitudinally followed were found to have spontaneous improvement or stabilization of hemodynamic status [15]. This was followed by a study 5 years later that described improvement in LVEF and resolution of HF in 11 of 97 patients with chronic congestive HF [16]. In this case series, the majority of patients (10/11) had a history of chronic alcoholism and abstained from alcohol use during follow-up, suggesting that excessive alcohol exposure may be a reversible etiology of HF.

The first study to formally characterize HFimpEF was a single-center, retrospective cohort study of 358 patients with HFrEF reported in 2011 [10, 11•]. In this study, the prevalence of HFimpEF (defined as a baseline LVEF ≤ 40% with subsequent LVEF > 40% at 9 months of follow-up) was 34%. It is interesting to note that 70% of patients who were initially classified as having HFpEF turned out to instead have HFimpEF, suggesting that many cases of HFimpEF may be misclassified as HFpEF or HFmrEF. Importantly, patients with HFimpEF were clinically distinct from those with chronic HFpEF despite having a similar LVEF, being younger and less likely to have comorbid conditions such as atrial fibrillation, hypertension, and diabetes. On the other hand, patients with HFimpEF were similar to those with HFrEF, though the former were younger and less likely to have coronary artery disease. With regard to medications, patients with HFimpEF and those with HFrEF were equally likely to use beta-blockers and RAS inhibitors, though patients with HFimpEF had lower use of loop diuretics and MRAs. Overall, patients with HFimpEF were found to have milder HF symptoms and lower rates of hospitalization compared with patients with HFrEF or HFpEF, providing evidence for HFimpEF as a distinct clinical entity.

The existence of HFimpEF as a clinically distinct sub-group of HF with improved clinical outcomes—including cardiovascular events and mortality—has been confirmed by subsequent studies, as summarized in Table 1 [7, 11•, 17]. Most recently, a retrospective cohort study analyzing data from 7,948 patients with HFrEF enrolled in the MECKI (Metabolic Exercise Cardiac Kidney Indexes) score database found that, compared with persistent HFrEF, HFimpEF (defined as baseline LVEF ≤ 40% with follow-up LVEF > 40% but < 50%) was associated with a more favorable prognosis, including reduced cardiovascular and overall mortality, better hemodynamic and neurohormonal profiles, and improved exercise performance [18]. Notably, this is despite patients with HFimpEF being less likely to be treated with a beta-blocker, MRA, or loop diuretic. Notwithstanding the improved prognosis, patients with HFimpEF were still at relatively high risk for long-term adverse events, highlighting the need for continual monitoring and treatment of these patients, especially those with mere partial recovery of LV function. Taken together, these findings suggest that although HFimpEF has improved prognosis compared with HFrEF, it still carries residual risk of adverse events.

## Predictors of LVEF improvement and reverse remodeling

**Fig. 1:**
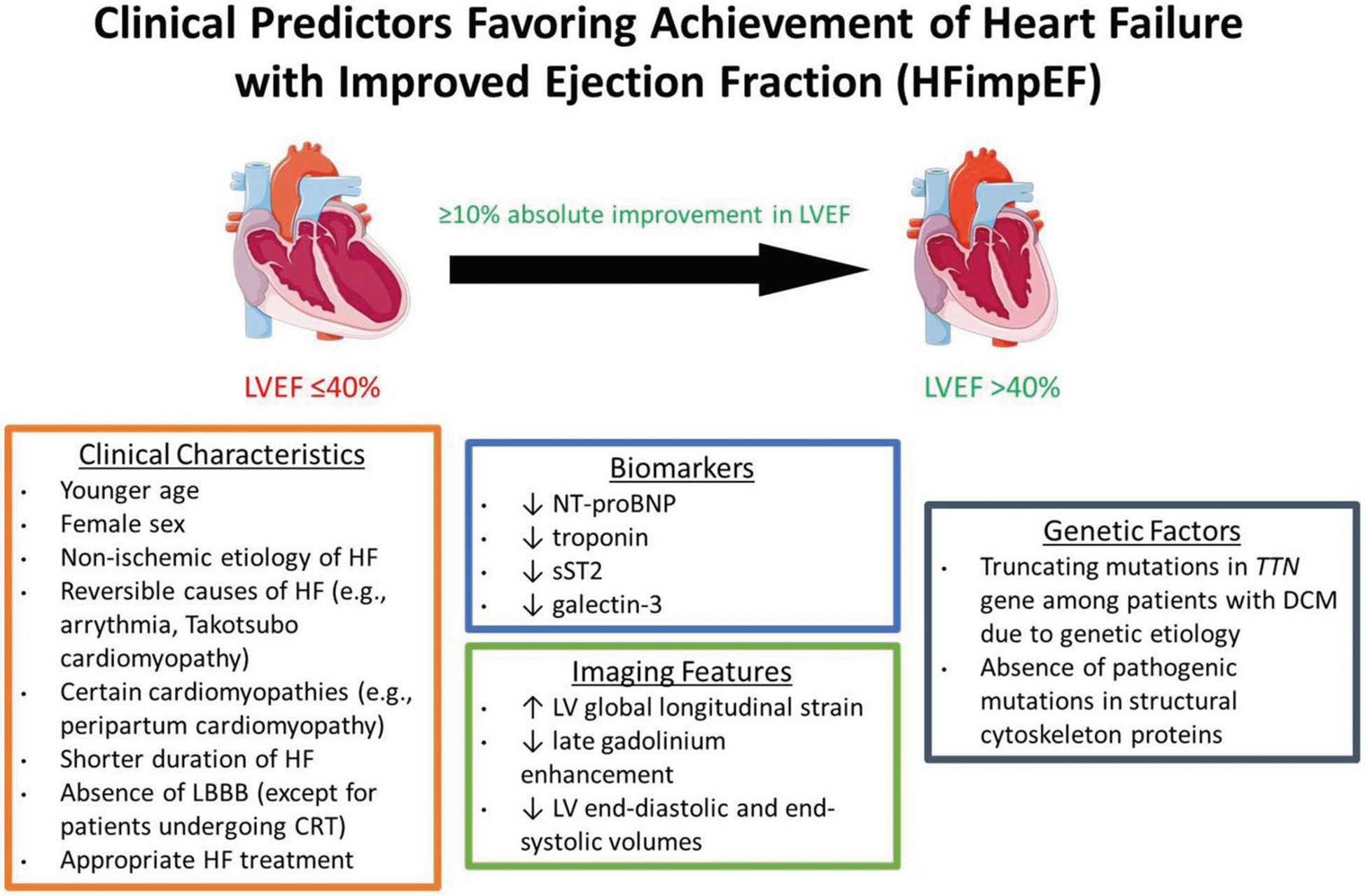
Clinical predictors favoring achievement of heart failure with improved ejection fraction Figure created using Servier Medical Art (licensed under CC BY 4.0, https://creativecommons.org/licenses/by/4.0/), based on papers by Wilcox et al. [11•] and Aimo et al. [35]. Abbreviations: HFimpEF, heart failure with improved ejection fraction; LVEF, left ventricular ejection fraction; HF, heart failure; LBBB, left bundle branch block; CRT, cardiac resynchronization therapy; NT-proBNP, aminoterminal pro-B-type natriuretic peptide; sST2, soluble suppression of tumorigenicity 2; LV, left ventricular; *TTN*, titin; DCM, dilated cardiomyopathy.

Clinical predictors of improved myocardial function and reverse remodeling include clinical characteristics, blood and imaging biomarkers, and genetic profiles (Fig. 1); these are discussed in turn below.

### Clinical characteristics

Over the past decade, several studies have shed light on the clinical characteristics associated with achieving LVEF improvement and HFimpEF (Table 2). While these studies have often used different study designs and clinical definitions for improvement of LVEF, their findings are largely concordant. Specifically, improvement of LVEF following medical therapy in HFrEF has been associated with younger age, female sex, absence of history of myocardial infarction, non-ischemic etiology of HF, reversible causes of HF or specific forms of cardiomyopathy, shorter duration of HF, and appropriate HF treatment regimen [5, 6, 8, 9, 11•, 14, 19–23]. Improvement of LVEF may also be more likely to occur in patients who have less severe baseline neurohormonal, hemodynamic, and biomarker profiles [6]. Notably, left bundle-branch block (LBBB) has a complicated relationship with LVEF improvement. While the presence of LBBB is an unfavorable prognostic factor in patients receiving GDMT only, it is a favorable prognostic factor in patients undergoing cardiac resynchronization therapy (CRT), presumably because LBBB reduces LVEF via LV dyssynchrony, which can be reversed with CRT [9, 11•, 19, 20].

The specific etiology of HFrEF appears to be an especially salient predictor of LVEF improvement or even recovery [24]. While LVEF recovery is less likely to occur in the setting of ischemia-related HF, it is more likely to occur in the setting of reversible causes of HFrEF such as arrhythmias (e.g., tachycardia-associated cardiomyopathy), endocrine disorders (e.g., thyroid diseases), stress-induced (or “Takotsubo”) cardiomyopathy, and certain toxic exposures (e.g., alcohol, the chemotherapy agent trastuzumab) that are managed appropriately [11•, 19]. Moreover, certain cardiomyopathies appear to be associated with greater likelihood of recovery, particularly those of an acute inflammatory nature, such as acute lymphocytic myocarditis and peripartum cardiomyopathy [11•]. Indeed, in the Pregnancy-Associated Cardiomyopathy study, a prospective cohort study that followed 100 patients with peripartum cardiomyopathy for 12 months post-partum, 72% of patients experienced complete recovery of LVEF to ≥ 50% [25]. These observations suggest that the specific etiology of HFrEF is a key determinant of prognosis.

### Blood biomarkers

Established and emerging biomarkers have tremendous potential to portend myocardial reverse remodeling and LVEF improvement, though no specific biomarker thresholds have been established to define HFimpEF. The most well characterized biomarker to date is N-terminal pro-B-type natriuretic peptide (NT-proBNP), which reflects the degree of myocyte stretch and concomitant wall stress [26, 27]. Several studies have demonstrated that reduced levels of NT-proBNP are associated with reverse remodeling and, in turn, improved prognosis [11•]. The PROTECT trial (Use of NT-proBNP Testing to Guide Heart Failure Therapy in the Outpatient Setting) compared NT-proBNP-guided therapy (with a goal of ≤ 1,000 pg/ml) with standard of care management in 151 patients with HFrEF and found that NT-proBNP-guided therapy reduced the primary endpoint of total cardiovascular events over a mean follow-up of 10 months, as well as improved LVEF along with LV end-systolic and end-diastolic volumes [28]. Notably, patients who failed to achieve NT-proBNP levels of ≤ 1,000 pg/mL experienced less or no improvement of echocardiographic measures compared with those who did [27, 29]. In accordance with this finding, a multivariable analysis of 116 patients enrolled in PROTECT trial found that higher NT-proBNP levels at study completion were associated with increased risk of future adverse myocardial remodeling based on echocardiographic measures [30]. In contrast with the PROTECT trial, the GUIDE-IT trial (Guiding Evidence Based Therapy Using Biomarker Intensified Treatment in Heart Failure) failed to demonstrate that routine NT-proBNP-guided therapy (with a goal of < 1,000 pg/mL) was superior to usual care in high-risk patients with HFrEF with respect to the primary composite outcome of time-to-first HF hospitalization and cardiovascular mortality [31]. Yet, this conflicting finding is presumably because both groups in the GUIDE-IT trial experienced similar reductions in NT-proBNP levels, unlike in the PROTECT trial. Indeed, a pre-specified echocardiographic analysis of the GUIDE-IT trial that included 124 patients from both groups found that reduction of NT-proBNP to < 1,000 pg/mL after 12 months of follow-up was associated with increased LVEF and decreased LV volumes [32]. Notably, the magnitude of reduction in NT-proBNP levels was proportional to the degree of reverse remodeling, with each decrement in NT-proBNP of 1,000 pg/mL corresponding to an increase in LVEF of 6.7% and a reduction in LV end-diastolic and end-systolic volumes by 15.7 ml/m^2^ and 17.3 ml/m^2^, respectively. Just as important, patients who achieved NT-proBNP < 1,000 pg/ml were less likely to experience adverse events. Findings from these two trials are corroborated by an analysis of the PARADIGM-HF trial demonstrating that reduction of NT-proBNP levels to ≤ 1,000 pg/ml was associated with reduced risk of cardiovascular mortality or HF hospitalization irrespective of treatment group [33]. Additionally, a secondary analysis of the REDEAL HF trial found that elevated baseline NT-proBNP levels (> 1,153 pg/mL) was the strongest independent risk factor of future cardiovascular events among patients with HFrEF both with or without improved LVEF [34]. Taken together, these findings suggest that NT-proBNP can serve as a biomarker to gauge the extent of reverse remodeling and, in turn, the prospects for improved prognosis. Notably, lower NT-proBNP levels reflect improved myocardial structure and function regardless of whether drug or device therapy is being used [35, 36], albeit no lower threshold for NT-proBNP has been identified yet.

Along with NT-proBNP, other biomarkers that are associated with different pathophysiologic mechanisms in HFrEF may offer independent insight into prognosis, including cardiac troponin, which reflects myocardial damage; soluble suppression of tumorigenesis-2 (sST2), which reflects inflammation; and galectin-3, which reflects fibrosis [11•, 35]. A multivariable model that incorporated a host of biomarkers associated with various pathophysiologic mechanisms in chronic HF was capable of improving risk stratification and prediction of adverse outcomes when used in tandem with the Seattle Heart Failure Model [37]. Another study identified that sST2 was the only studied biomarker that was independently associated with LV reverse remodeling and LVEF recovery at 12 months of follow-up in a cohort of 304 patients with HFrEF, and that a multivariable model incorporating this biomarker was capable of accurately assessing LV reverse remodeling in multiple other cohorts and predicting mortality at up to 4 years [38, 39]. Emerging biomarkers such as various miRNAs and extracellular matrix proteins may also inform prognosis, though further research is needed [27, 35].

### Imaging modalities

Imaging modalities such as echocardiography and cardiac magnetic resonance imaging (MRI) are integral tools for assessing whether reverse remodeling has occurred and LVEF function has improved. Echocardiographic changes associated with reverse remodeling and improved prognosis include reduced LV end-diastolic and end-systolic volumes, reduced left atrial volume parameters, improved mitral regurgitation, and absence of right ventricular dysfunction [11•, 35, 39, 40]. In addition to these parameters, a measure of intrinsic LV contractility known as LV global longitudinal strain (GLS) may predict reverse remodeling in some patients with HFrEF [11•]. A recent study found that a higher baseline absolute GLS (> 8%) was associated with greater achievement of HFimpEF among patients with non-ischemic HFrEF and larger left ventricular dimensions [11•, 41]. In a separate study, normal values for GLS (> 16%) were associated with better durability of LVEF recovery, though it should be acknowledged that GLS rarely normalizes in patients with HFimpEF [11•, 42].

In recent years cardiac MRI has been increasingly adopted into clinical practice, as it provides greater resolution and reproducibility than echocardiography, not to mention better insights into the etiology of HF [11•, 19]. In particular, cardiac MRI can assess the degree of myocardial fibrosis via late gadolinium enhancement (LGE), with less extent of LGE being associated with a lower degree of fibrosis and, therefore, a higher probability of reverse remodeling [11•, 19, 35, 43, 44]. Indeed, absence of LGE has been shown to have a high positive predictive value and specificity for reverse remodeling and concomitant LVEF recovery, irrespective of baseline LVEF and LV volumes [19, 35, 44, 45]. In addition to LGE, the degree of myocardial fibrosis and extracellular volume (ECV) can also be assessed by a different cardiac MRI technique known as T1 mapping, which appears to provide additional insight into myocardial fibrosis and, therefore, may be combined with LGE to enhance prediction of reverse remodeling [11•, 35, 46].

### Genetic analyses

The genetic underpinnings of HF may offer insight into the likelihood of recovery, with specific variant alleles being associated with better or worse prognosis. For example, a mutation resulting in downregulation of the *CDCP1* gene (which drives proliferation of cardiac fibroblasts) was found to be associated with improvement of myocardial function in patients with dilated cardiomyopathy (DCM), presumably via attenuation of fibrosis [47]. In considering the etiology of DCM, genetic causes may be associated with lower chance of recovery compared with non-genetic causes [19, 48]. Yet among patients with genetic causes of DCM, specific mutations may portend a greater likelihood of improvement in LVEF following GDMT. A notable example is the *TTN* gene, which encodes the sarcomere protein titin [11•]. Truncating mutations in *TTN* represent the most common genetic etiology of DCM, accounting for 15–25% of cases, and *TTN* variants have been found to be associated with a relatively mild form of HF that is more amenable to treatment and recovery than other genetic etiologies [11•, 49–51]. Conversely, certain genetic mutations in Lamin A/C (*LMNA*), *SCN5A*, desmoplakin (*DSP*), and filamin C (*FLNC*) are associated with life-threatening arrythmias that pose continual risk of sudden cardiac death, even once LVEF has recovered [11•].

## New mechanistic insights into reverse remodeling in HFimpEF

The pathophysiology of HFrEF is driven by LV remodeling, which entails increased activation of the sympathetic nervous system and the renin–angiotensin–aldosterone (RAAS) system with concomitant pathologic changes in myocardial structure and geometry (i.e., increased LV dimensions, volume, and mass leading to transition from an elliptical-shaped heart to one that is spherical), resulting in impaired LV function [24, 35]. At the molecular and cellular level, pathophysiologic mechanisms underlying LV remodeling include cardiomyocyte hypertrophy along with disruption of cardiomyocyte function (e.g., increased fetal gene expression, decreased excitation–contraction coupling, altered activity of cytoskeletal proteins, beta-adrenergic desensitization); reduced survival and increased apoptosis of cardiomyocytes; alterations to the extracellular matrix (e.g., increased fibrosis, decreased angio-genesis); and perturbations to myocardial metabolism, specifically reduced metabolism of fatty acids with increased reliance on glucose and ketone bodies [11•, 24]. Amelioration of these pathophysiologic mechanisms, a phenomenon known as reverse remodeling, can occur either spontaneously or in response to therapy with GDMT, cardiac resynchronization therapy, or an LV assist device (LVAD), resulting in partial or complete normalization of LV structure and function along with improved prognosis [11•]. The primary driver of the reverse remodeling process is thought to be reduced biomechanical load on the failing heart, given that myocardial unloading appears to induce physiological pathways involved in reverse remodeling [11•, 52]. A comprehensive and detailed account of the physiologic mechanisms underlying reverse remodeling is beyond the scope of this review and can be found in other review articles [53–55]. Here, we limit our discussion to the most recent findings and key concepts.

Recent studies have sought to harness various unbiased molecular techniques to identify differences in the molecular profile between patients who respond to therapy and experience improvement of myocardial function versus those who do not. A prospective study employing unbiased transcriptomics and proteomics in 93 patients with HF who underwent LVAD implantation found that 29 transcripts and 93 phospho-peptides differentiated patients who responded to LVAD therapy versus non-responders [56]. Upon further analysis it was found that these differences in molecular profile reflected differential regulation of a couple of key pathways, namely the cell cycle and the extracellular matrix along with focal adhesions. In another study, single-nucleus RNA sequencing was performed in patients with HF who underwent LVAD implantation, revealing cell-specific transcriptional differences between patients who experienced recovery of myocardial function versus those who did not [57]. These transcriptional differences were largely localized to macrophages and fibroblasts, with inflammatory signatures being inversely associated with likelihood of recovery.

At the molecular level, reverse remodeling does not appear to simply be a reversal of the forward, pathologic remodeling process [11•, 58]. Indeed, transcriptional profiling of hearts that have undergone reverse remodeling has revealed that some gene networks continue to remain dysregulated despite improvements in myocardial structure and function, along with upregulation of other gene networks that are not typically expressed in normal, non-failing hearts [11•, 58, 59]. Hence, reverse remodeling does not result in complete normalization of the pathologic molecular changes associated with forward remodeling but rather merely engenders a “less pathologic steady state” that enables the heart to restore LV function under normal physiologic conditions [11]. Because this adaptive myocardial steady state has reduced contractile reserve, it is susceptible to deterioration in the setting of physiologic or environmental stressors, which likely explains why some patients who experience reverse remodeling and recovery of LV function nonetheless remain at risk of relapse and adverse events [11•, 59].

## Clinical predictors of relapse versus sustained remission

While achieving reverse remodeling and LVEF improvement appears to be associated with better prognosis, the natural history of HFimpEF remains poorly understood. Whether a given patient with HFimpEF is likely to relapse or sustain remission is of particular clinical interest, as this question presumably dictates the extent of monitoring and treatment that the patient should receive. Even among patients who continue GDMT, the rate of deterioration or relapse can be notable, ranging from 28%−80% depending on the study cohort and design [60–62]. A retrospective cohort study of 408 patients with DCM on optimal GDMT found that 9% of patients had recovery of LVEF to ≥ 50% and normalization of LV end-diastolic dimensions at roughly 8.6 years of follow-up, but that among these patients over a third (37%) subsequently experienced deterioration of LVEF to < 50%, with 5% requiring heart transplant or dying at 15 years of follow-up [61]. Another retrospective cohort study using the currently endorsed definition for HFimpEF found that although 57% of patients with HFrEF achieved HFimpEF at a median follow-up of 13 months (460 of 800 patients), 41% of these patients subsequently experienced recurrent LVEF ≤ 40%, with increased risk of the primary composite endpoint of all-cause death, heart transplantation, or LVAD placement [63]. Similarly, a retrospective cohort study of 2,319 patients with non-ischemic HFrEF with LVEF < 35% found that among 465 patients (20%) who experienced recovery of LVEF to ≥ 50% at a mean follow-up of 12 months, 50% subsequently experienced deterioration of LVEF to < 50% within 3.5 years, and 80% experienced deterioration within 7 years [60]. Importantly, serial echocardiographic LVEF measurements in this study were predictive of long-term prognosis: a longer duration of sustained LVEF recovery was associated with better survival, with patients who sustained recovery for longer than 5 years having a 10-year survival rate of 83%. This is corroborated by an analysis of the BEST trial that revealed distinct patterns of LVEF improvement and found that sustained improvement at 12 months of follow-up was associated with better event-free survival from all-cause death or HF hospitalization over a mean follow-up of 49 months compared with transient improvement [64].

Because greater durability of remission is associated with improved prognosis, there has been increasing research seeking to identify the clinical predictors of deterioration versus sustained improvement of LVEF among patients with HFimpEF. A recent retrospective cohort study analyzed 7,070 patients with echocardiographic diagnosis of HFimpEF and found that characteristics associated with sustained improvement of LVEF at a median follow-up of 15.9 months (occurred in 37.6% patients) included White race and continued use of a RAS inhibitor, while characteristics associated with deterioration included male sex, atrial fibrillation or flutter, coronary artery disease, history of myocardial infarction, presence of an implantable cardioverter-defibrillator, and use of loop diuretics [65]. Deterioration was also more common in patients who were in the lowest quartile for LVEF or highest quartile for LV end-diastolic or end-systolic volume at baseline. Another retrospective study analyzed 133 patients who had experienced recovery of LVEF from < 40% to ≥ 53% and found that 28% of these patients experienced deterioration of LVEF to < 40% at 3 years of follow-up, with the strongest predictors of deterioration being baseline diuretic dose and levels of natriuretic peptide at the time of LVEF recovery [62]. Using these two factors in combination with New York Heart Association functional class, the investigators were able to construct a predictive model that accurately stratifies patients with LVEF recovery based on risk of mortality. Perhaps one of the most promising clinical predictors of HFimpEF prognosis is the echocardiographic measure GLS: a retrospective cohort study of 289 patients with HFimpEF found that a higher absolute value of GLS was associated with reduced likelihood of LVEF deterioration over a median follow-up of 53 months, not to mention reduced risk of the primary endpoint of time to first occurrence of cardiovascular mortality or adverse HF events, with each 1% increase in absolute GLS being associated with a 10% decreased risk of the primary endpoint after multivariable adjustment [66]. Other factors that have been associated with deterioration of LVEF and adverse outcomes include longer duration from diagnosis of HFrEF to recovery of LVEF, larger LV end-diastolic dimensions at diagnosis of HFrEF, diastolic dysfunction as assessed by echocardiography, older age, and presence or development of comorbidities such as diabetes, atrial fibrillation, and coronary artery disease [11•, 63, 67–69].

## Conclusions and future directions

HFimpEF is associated with improved prognosis compared with persistent HFrEF, albeit with continual risk of adverse events and risk of relapse. Several clinical predictors of achieving HFimpEF have been identified, including clinical characteristics, biomarkers, imaging features, and genetic factors. Based on insights from clinical trials, observational studies, and mechanistic studies, it is now known that many cases of HFimpEF entail HFrEF in remission as opposed to recovery of HFrEF.

Although much has been learned about this clinical entity in recent years, there remain several challenges and knowledge gaps. There is significant variability among studies regarding criteria used to define HFimpEF, making it difficult to reconcile study findings; to avoid this issue going forward, future studies should use the current consensus definition for HFimpEF, as described above. Additionally, there is a need for further prospective cohort studies outlining the natural history of HFimpEF, including the risk of relapse versus sustained remission over time, as well as the clinical predictors of relapse versus sustained remission. These studies should also seek to identify whether distinct subgroups of HFimpEF might have different clinical trajectories and prognoses, such as patients with partial recovery of LVEF versus those with full recovery (LVEF 41–49% versus LVEF ≥ 50%, respectively). In addition to differentiating patients based on conventional imaging-based measures such as LVEF or left ventricular dimensions or volumes, blood biomarkers such as NT-proBNP or other multi-omics could be used to tease apart distinct subgroups. Also warranting further investigation is the question of whether biological signatures from myocardial tissue samples or novel imaging modalities (such as molecular imaging or novel techniques examining subclinical abnormalities in myocardial tissue architecture) could be used to distinguish subgroups of patients, as these approaches could guide safe de-escalation of GDMT. Together, these studies will provide greater insight into the biological underpinnings and natural history of HFimpEF, thereby better informing management of this condition.

## Figures and Tables

**Table 1.** Key studies characterizing HFimpEF

Study Title	Study Design	Definition used for HFimpEF	Prevalence of HFimpEF	Key Findings
Heart Failure With Recovered Ejection Fraction: A Distinct Clinical Entity [10]	Single-center, retrospective cohort study of 358 patients at Bringham and Women’s Hospital	Baseline LVEF ≤ 40% with subsequent LVEF > 40% at 9 months of follow-up	34%	Patients with HFimpEF were similar to those with HFrEF, albeit the former were younger and less likely to have coronary artery disease. Notably, both groups of patients were equally likely to use beta-blockers and RAS inhibitors, though patients with HFimpEF had lower use of loop diuretics and MRAs. In contrast, patient with HFimpEF and those with HFpEF were less similar, with the former being younger and less likely to have conditions such as atrial fibrillation, hypertension, and diabetes. Patients with HFimpEF had milder HF symptoms and lower rates of hospitalization compared with patients with HFrEF or HFpEF.
Heart Failure with Recovered Ejection Fraction: Clinical Description, Biomarkers, and Outcomes [17]	Multi-center, prospective cohort study of 1,821 chronic HF patients enrolled into the Penn Heart Failure Study (PHFS)	LVEF ≥ 50% upon enrollment into PHFS but a previous LVEF < 50%	~10%	Patients with HFimpEF had lower levels of adverse biomarkers (e.g., troponin I, brain natriuretic factor, soluble fms-like tyrosine kinase receptor-1) than those with either HFrEF or HFpEF, albeit many patients with HFimpEF still had an abnormal biomarker profile. Patients with HFimpEF had better clinical outcomes than those with HFrEF or HFpEF over a maximum follow-up of roughly 9 years: relative to the HFimpEF group, the unadjusted hazard ratio for the composite terminal event consisting of all-cause death, transplantation, or ventricular assist device placement in HFrEF and HFpEF was 4.1 (95% CI, 2.4–6.8; P < 0.001) and 2.3 (95% CI, 1.2–4.5; P = 0.013), respectively (with similar results observed in adjusted models). Notably, the better outcomes observed in patients with HFimpEF compared with HFrEF were not due to the former having higher rates of GDMT, with in fact lower utilization of MRAs among patients with HFimpEF. Patients with HFimpEF also had lower rates of hospitalization than those with HFrEF, though they still experienced a significant number of hospitalizations, with rates similar to those with HFpEF.
Characteristics and Outcomes of Adult Outpatients With Heart Failure and Improved or Recovered Ejection Fraction [7]	Single-center, retrospective cohort study of 2,166 HF patients at Emory University	LVEF > 40% with previously documented LVEF ≤ 40%	16.2%	After 3 years of follow-up, patients with HFimpEF had lower adjusted all-cause mortality rates, with a rate of 4.8% compared with 16.3% and 13.2% in patients with persistent HFrEF or HFpEF, respectively. Patients with HFimpEF also had lower risk for HF hospitalizations (adjusted rate ratio, 0.48; 95 CI, 0.30–0.76; P = 0.002). Notably, use of RAS inhibitors and beta-blockers was similar among patients with HFimpEF and those with HFrEF, with lower use of loop diuretics in the former group.
Heart failure patients with improved ejection fraction: Insights from the MECKI score database [18]	Retrospective cohort study analyzing data from 7,948 patients with HFrEF enrolled in the MECKI (Metabolic Exercise Cardiac Kidney Indexes) score database	Baseline LVEF ≤ 40% with follow-up LVEF > 40% but < 50% at a median follow-up of 1,490 days Notably, the authors chose to exclude patients with recovery of LVEF to ≥ 50% (n = 403) from the HFimpEF group because they argued that patients with complete recovery of LVEF are likely phenotypically distinct from those with partial recovery.	19%	Compared with patients with persistent HFrEF (n = 6017), those who achieved HFimpEF (n = 1504) had a more favorable prognosis, including reduced cardiovascular and overall mortality, better hemodynamic and neurohormonal profiles, and improved exercise performance. Notably, use of a RAS inhibitor was similar among patients with HFimpEF and those with persistent HFrEF, though patients with HFimpEF were less likely to be treated with a beta-blocker, MRA, or loop diuretic. Despite improved prognosis, patients with HFimpEF were still at relatively high risk for adverse events.
Prevalence and Prognosis of HFimpEF Developed From Patients With Heart Failure With Reduced Ejection Fraction: Systematic Review and Meta-Analysis [70]	Systematic review and meta-analysis of 9 observational studies (5 prospective and 4 retrospective) collectively entailing 9,491 patients with HF	Variable definitions depending on specific study: 5 studies: previous LVEF ≤ 40% with subsequent LVEF >40% 2 studies: previous LVEF < 50% with subsequent LVEF ≥ 50% 1 study: previous LVEF < 40% with subsequent LVEF ≥ 50% 1 study: previous LVEF < 45% with subsequent LVEF ≥ 45% Mean follow-up of 3.8 years across all studies	22.64%	Compared with HFrEF, HFimpEF was associated with lower risk of mortality (adjusted hazard ratio, 0.44; 95% CI, 0.33–0.60) and cardiac hospitalization (hazard ratio, 0.40; 95% CI, 0.20–0.82). HFimpEF was also associated with lower risk of mortality (adjusted hazard ratio, 0.42; 95% CI, 0.32–0.55) and cardiac hospitalization (hazard ratio, 0.73; 95% CI, 0.58–0.92) compared with HFpEF.

*HFimpEF* heart failure with improved ejection fraction, *LVEF* left ventricular ejection fraction, *HF* heart failure, *HFrEF* heart failure with reduced ejection fraction, *HFpEF* heart failure with preserved ejection fraction, *PHFS* Penn Heart Failure Study, *RAS* renin angiotensin system, *MECKI* Metabolic Exercise Cardiac Kidney Indexes, *GDMT* guideline-directed medical therapy

**Table 2.** Studies identifying clinical characteristics associated with improvement of myocardial function among patients with HF

Study Title	Study Design	Definition used for Improvement of Myocardial Function	Clinical Characteristics Associated with Improvement	Other Key Findings
Factors associated with improvement in ejection fraction in clinical practice among patients with heart failure: Findings from IMPROVE HF [14]	Multi-site, prospective study using data from IMPROVE HF (Registry to Improve the Use of Evidence-Based Heart Failure Therapies in the Outpatient Setting) examining 3,994 patients with HFrEF	Improvement of LVEF by > 10% at 24 months of follow-up	Female sex, lack of history of myocardial infarction, and nonischemic etiology of HF	28.6% of patients experienced improvement of LVEF
Recovered heart failure with reduced ejection fraction and outcomes: a prospective study [9]	Single-center, prospective cohort study of 1,057 HF patients from a university hospital in Spain	Baseline LVEF < 45% with improvement to ≥ 45% at 1 year of follow-up	Younger age, female sex, shorter duration of HF, non-ischemic etiology of HF, and absence of left bundle-branch block	25% of patients experienced improvement of LVEF, with an average increase in LVEF of 21.1% Notably, at least 12% of patients who experienced improvement had a reversible cause of HF such as alcoholic cardiomyopathy or tachycardia-induced cardiomyopathy. At an average follow-up of 3.1 years, patients with improvement of LVEF had lower risk for the primary composite endpoint of cardiovascular death or HF hospitalization, as well as for all-cause death.
Frequency, predictors, and prognosis of ejection fraction improvement in heart failure: an echocardiogram-based registry study [5]	Retrospective cohort study of 10,641 HF patients living in Alberta	Baseline LVEF ≤ 40% with an absolute improvement in LVEF of ≥ 10% at a minimum of 6 months of follow-up	Younger age, female sex, and comorbid conditions such as hypertension and atrial fibrillation (possibly because comorbidities may be associated with more aggressive management of HF)	37.6% of patients experienced improvement of LVEF
Longitudinal Changes in Ejection Fraction in Heart Failure Patients With Preserved and Reduced Ejection Fraction [8]	Retrospective cohort study of a community population in Minnesota consisting of 1,233 HF patients	Improvement in serial LVEF measures	Younger age, female sex, lack of history of coronary artery disease, and treatment with HF medications	Improvement in serial LVEF measures was associated with increased survival
Clinical, Echocardiographic, and Longitudinal Characteristics Associated With Heart Failure With Improved Ejection Fraction [22]	Single-center retrospective cohort study consisting of 1,307 patients with HFrEF	Development of HFimpEF, as defined by a baseline LVEF < 40% with improvement of LVEF by at least 10% to > 40% at a minimum follow-up of 3 months	Female sex and presence of co-morbidities (possibly because comorbidities may be associated with more aggressive management of HF)	38.7% of patients developed HFimpEF at a median follow-up of 16.3 months The HFimpEF group had better survival compared with the persistent HFrEF group (p < 0.001).
Heart Failure With Improved Ejection Fraction: Clinical Characteristics, Correlates of Recovery, and Survival: Results From the Valsartan Heart Failure Trial [6]	Secondary analysis of the Valsartan Heart Failure Trial consisting of 3,519 patients with HFrEF	Baseline LVEF < 35% with improvement to > 40% at 1 year of follow-up	Lack of ischemic heart disease, treatment with an intense HF medication regimen	9.1% of patients experienced improvement of LVEF Patients who experienced improvement tended to have less severe measures for their neurohormonal, hemodynamic, and biomarker profiles
Improvement of ejection fraction and mortality in ischemic heart failure [23]	Secondary analysis of the Surgical Treatment for Ischemic Heart Failure trial consisting of 618 HFrEF patients with coronary artery disease	Baseline LVEF ≤ 35% with improvement to ≥ 10% at 24 months of follow-up	Lack of history of myocardial infarction	Roughly 18% of patients experienced improvement of LVEF Improvement of LVEF by ≥ 10% was independently associated with lower risk of mortality (hazard ratio, 0.61; 95%CI, 0.44–0.84; p = 0.004).

*HF* heart failure, *IMPROVE HF* Registry to Improve the Use of Evidence-Based Heart Failure Therapies in the Outpatient Setting, *HFrEF* heart failure with reduced ejection fraction, *HF* heart failure, *LVEF* left ventricular ejection fraction, *HFimpEF* heart failure with improved ejection fraction

## Opinion statement

Among patients with heart failure with reduced ejection fraction (HFrEF), guideline-directed medical therapy (GDMT) and device therapies offer significant benefit, particularly among those with persistently reduced left ventricular ejection fraction (LVEF) and or elevated levels of natriuretic peptides. Over the years there has been increasing awareness that many of these patients in fact experience partial or complete recovery of LVEF along with improved prognosis, a phenomenon termed heart failure with improved ejection fraction (HFimpEF). Recent findings suggest that HFimpEF is likely a heterogeneous clinical entity that comprises distinct subgroups of patients with varying underlying pathophysiologic processes and differential prognoses. Of notable interest is whether a given patient with HFimpEF is merely in remission or has fully recovered with respect to the pathophysiologic mechanisms underlying their condition, as this presumably guides management of that patient. Given that conventional imaging-derived measures such as LVEF and left ventricular volumes fail to distinguish between mere remission and fully recovery, there is a need for clinical tools that more accurately and precisely reflect the unique underlying myocardial biology of a given patient. We argue that such clinical tools could include biomarkers such as N-terminal pro-B-type natriuretic peptide (NT-proBNP), which reflects myocardial myocyte stress. This calls for future studies investigating whether biomarkers such as NT-proBNP can distinguish between patients with HFimpEF who are merely in remission versus those who have fully recovered, as well as whether this can be leveraged to tailor management for these patients.

## Abbreviations

DCM: Dilated cardiomyopathy
GDMT: Guideline-directed medical therapy
GLS: Global longitudinal strain
HF: Heart failure
HFimpEF: Heart failure with improved ejection fraction
HFmrEF: Heart failure with mildly reduced ejection fraction
HFpEF: Heart failure with preserved ejection fraction
HFrecEF: Heart failure with recovered ejection fraction
HFrEF: Heart failure with reduced ejection fraction
LBBB: Left bundle-branch block
LGE: Late gadolinium enhancement
LVAD: LV assist device
LVEF: Left ventricular ejection fraction
MRA: Mineralocorticoid receptor antagonist
MRI: Magnetic resonance imaging
NT-proBNP: N-terminal pro-B-type natriuretic peptide
RAAS: Renin-angiotensin-aldosterone
RAS: Renin-angiotensin
SGLT2i: Sodium-glucose cotransporter-2 inhibitor
sST2: Soluble suppression of tumorigenesis-2

## References

[1] Bowen RES, Graetz TJ, Emmert DA, Avidan MS. Statistics of heart failure and mechanical circulatory support in 2020. Ann Transl Med. 2020;8:827–827. 32793672 10.21037/atm-20-1127 PMC7396255

[2] Savarese G, Lund LH. Global public health burden of heart failure. Card Fail Rev. 2017;3:7–11. 28785469 10.15420/cfr.2016:25:2 PMC5494150

[3] Heidenreich PA, Bozkurt B, Aguilar D, 2022 AHA/ACC/HFSA Guideline for the Management of Heart Failure: Executive Summary: A Report of the American College of Cardiology/American Heart Association Joint Committee on Clinical Practice Guidelines. J Am Coll Cardiol. 2022;79:1757–80. 35379504 10.1016/j.jacc.2021.12.011

[4] Docherty KF, Bayes-Genis A, Butler J, Coats AJS, Drazner MH, Joyce E, Lam CSP. The four pillars of HFrEF therapy: is it time to treat heart failure regardless of ejection fraction? Eur Heart J Suppl. 2022;24:L10–9. 36545228 10.1093/eurheartjsupp/suac113 PMC9762881

[5] Ghimire A, Fine N, Ezekowitz JA, Howlett J, Youngson E, McAlister FA. Frequency, predictors, and prognosis of ejection fraction improvement in heart failure: an echocardiogram-based registry study. Eur Heart J. 2019;40:2110–7. 31280320 10.1093/eurheartj/ehz233

[6] Florea VG, Rector TS, Anand IS, Cohn JN. Heart failure with improved ejection fraction: clinical characteristics, correlates of recovery, and survival. Circ Heart Fail. 2016;9:e003123. 10.1161/CIRCHEARTFAILURE.116.003123 27413037

[7] Kalogeropoulos AP, Fonarow GC, Georgiopoulou V, Burkman G, Siwamogsatham S, Patel A, Li S, Papadimitriou L, Butler J. Characteristics and Outcomes of Adult Outpatients With Heart Failure and Improved or Recovered Ejection Fraction. JAMA Cardiology. 2016;1:510–8. 27434402 10.1001/jamacardio.2016.1325

[8] Dunlay SM, Roger VL, Weston SA, Jiang R, Redfield MM. Longitudinal changes in ejection fraction in heart failure patients with preserved and reduced ejection fraction. Circ Heart Fail. 2012;5:720–6. 22936826 10.1161/CIRCHEARTFAILURE.111.966366 PMC3661289

[9] Lupón J, Díez-López C, de Antonio M, Domingo M, Zamora E, Moliner P, González B, Santes-mases J, Troya MI, Bayés-Genís A. Recovered heart failure with reduced ejection fraction and outcomes: a prospective study. Eur J Heart Fail. 2017;19:1615–23. 28387002 10.1002/ejhf.824

[10] Punnoose LR, Givertz MM, Lewis EF, Pratibhu P, Stevenson LW, Desai AS. Heart Failure With Recovered Ejection Fraction: A Distinct Clinical Entity. J Cardiac Fail. 2011;17:527–32. 10.1016/j.cardfail.2011.03.005 21703523

[11.•] Wilcox JE, Fang JC, Margulies KB, Mann DL. Heart Failure With Recovered Left Ventricular Ejection Fraction: JACC Scientific Expert Panel. J Am Coll Cardiol. 2020;76:719–34. 32762907 10.1016/j.jacc.2020.05.075

[12.••] Bozkurt B, Coats AJS, Tsutsui H, Universal definition and classification of heart failure: a report of the heart failure society of america, heart failure association of the european society of cardiology, japanese heart failure society and writing committee of the universal definition of heart failure. Eur J Heart Fail. 2021;23:352–80. 33605000 10.1002/ejhf.2115

[13.•] Halliday BP, Wassall R, Lota AS, Withdrawal of pharmacological treatment for heart failure in patients with recovered dilated cardiomyopathy (TRED-HF): an open-label, pilot, randomised trial. The Lancet. 2019;393:61–73. 10.1016/S0140-6736(18)32484-X PMC6319251 30429050

[14] Wilcox JE, Fonarow GC, Yancy CW, Factors associated with improvement in ejection fraction in clinical practice among patients with heart failure: Findings from IMPROVE HF. Am Heart J. 2012;163:49–56.e2. 22172436 10.1016/j.ahj.2011.10.001

[15] Figulla HR, Rahlf G, Nieger M, Luig H, Kreuzer H. Spontaneous hemodynamic improvement or stabilization and associated biopsy findings in patients with congestive cardiomyopathy. Circulation. 1985;71:1095–104. 3995705 10.1161/01.cir.71.6.1095

[16] Francis GS, Johnson TH, Ziesche S, Berg M, Boosalis P, Cohn JN. Marked spontaneous improvement in ejection fraction in patients with congestive heart failure. Am J Med. 1990;89:303–7. 2203261 10.1016/0002-9343(90)90342-b

[17] Basuray A, French B, Ky B, Vorovich E, Olt C, Sweitzer NK, Cappola TP, Fang JC. Heart failure with recovered ejection fraction. Circulation. 2014;129:2380–7. 24799515 10.1161/CIRCULATIONAHA.113.006855 PMC4053508

[18] Agostoni P, Pluchinotta FR, Salvioni E, Heart failure patients with improved ejection fraction: Insights from the MECKI score database. Eur J Heart Fail. 2023;25:1976–84. 37702313 10.1002/ejhf.3031

[19] Chen X, Wu M. Heart failure with recovered ejection fraction: Current understanding and future prospects. Am J Med Sci. 2023;365:1–8. 36084706 10.1016/j.amjms.2022.07.018

[20] Proclemer A, Muser D, Facchin D. What We Can Learn from “Super-responders.” Heart Fail Clin. 2017;13:225–32. 27886927 10.1016/j.hfc.2016.07.018

[21] Bermejo RA, Babarro EG, Canoa JNL, Román AV, Otero IG, Ayude MO, Vazquez PP, Rodríguez IG, Castro OD, Juanatey JRG. Heart failure with recovered ejection fraction: Clinical characteristics, determinants and prognosis. CARDIOCHUS-CHOP registry. Cardiol J. 2018;25:353–62. 28980289 10.5603/CJ.a2017.0103

[22] Romero E, Baltodano AF, Rocha P, Clinical, echocardiographic, and longitudinal characteristics associated with heart failure with improved ejection fraction. Am J Cardiol. 2024;211:143–52. 37923155 10.1016/j.amjcard.2023.10.086 PMC10869234

[23] Perry AS, Mann DL, Brown DL. Improvement of ejection fraction and mortality in ischaemic heart failure. Heart. 2021;107:326–31. 10.1136/heartjnl-2020-316975 32843496

[24] Kuttab JS, Kiernan MS, Vest AR. Epidemiology of “heart failure with recovered ejection fraction”: what do we do after recovery? Curr Heart Fail Rep. 2015;12:360–6. 26486630 10.1007/s11897-015-0274-4

[25] McNamara DM, Elkayam U, Alharethi R, Clinical outcomes for peripartum cardiomyopathy in north america. J Am Coll Cardiol. 2015;66:905–14. 26293760 10.1016/j.jacc.2015.06.1309 PMC5645077

[26] Yan CL, Grazette L. A review of biomarker and imaging monitoring to predict heart failure recovery. Front Cardiovasc Med. 2023;10:1150336. 10.3389/fcvm.2023.1150336 PMC10117884 37089891

[27] Motiwala SR, Gaggin HK. Biomarkers to predict reverse remodeling and myocardial recovery in heart failure. Curr Heart Fail Rep. 2016;13:207–18. 27726056 10.1007/s11897-016-0303-y

[28] Januzzi JL, Rehman SU, Mohammed AA, Use of amino-terminal pro–B-type natriuretic peptide to guide outpatient therapy of patients with chronic left ventricular systolic dysfunction. J Am Coll Cardiol. 2011;58:1881–9. 22018299 10.1016/j.jacc.2011.03.072

[29] Gaggin HK, Truong QA, Rehman SU, Characterization and prediction of natriuretic peptide “Nonresponse” during heart failure management: results from the probnp outpatient tailored chronic heart failure (PROTECT) and the NT-proBNP–assisted treatment to lessen serial cardiac readmissions and death (BATTLESCARRED) study. Congest Heart Fail. 2013;19:135–42. 23279139 10.1111/chf.12016

[30] Weiner RB, Baggish AL, Chen-Tournoux A, Improvement in structural and functional echocardiographic parameters during chronic heart failure therapy guided by natriuretic peptides: mechanistic insights from the ProBNP outpatient tailored chronic heart failure (PROTECT) study. Eur J Heart Fail. 2013;15:342–51. 23132825 10.1093/eurjhf/hfs180

[31] Felker GM, Anstrom KJ, Adams KF, Effect of natriuretic peptide-guided therapy on hospitalization or cardiovascular mortality in high-risk patients with heart failure and reduced ejection fraction: a randomized clinical trial. JAMA. 2017;318:713–20. 28829876 10.1001/jama.2017.10565 PMC5605776

[32] Daubert MA, Adams K, Yow E, NT-proBNP Goal achievement is associated with significant reverse remodeling and improved clinical outcomes in HFrEF. JACC: Heart Fail. 2019;7:158–68. 30611722 10.1016/j.jchf.2018.10.014

[33] Zile MR, Claggett BL, Prescott MF, Prognostic implications of changes in N-terminal pro-B-type natriuretic peptide in patients with heart failure. J Am Coll Cardiol. 2016;68:2425–36. 27908347 10.1016/j.jacc.2016.09.931

[34] Liu D, Hu K, Schregelmann L, Hammel C, Lengen-felder BD, Ertl G, Frantz S, Nordbeck P. Determinants of ejection fraction improvement in heart failure patients with reduced ejection fraction. ESC Heart Fail. 2023;10:1358–71. 36732921 10.1002/ehf2.14303 PMC10053299

[35] Aimo A, Gaggin HK, Barison A, Emdin M, Januzzi JL. Imaging, biomarker, and clinical predictors of cardiac remodeling in heart failure with reduced ejection fraction. JACC: Heart Fail. 2019;7:782–94. 31401101 10.1016/j.jchf.2019.06.004

[36] Fruhwald FM, Fahrleitner-Pammer A, Berger R, Early and sustained effects of cardiac resynchronization therapy on N-terminal pro-B-type natriuretic peptide in patients with moderate to severe heart failure and cardiac dyssynchrony. Eur Heart J. 2007;28:1592–7. 17298973 10.1093/eurheartj/ehl505

[37] Ky B, French B, Levy WC, Sweitzer NK, Fang JC, Wu AHB, Goldberg LR, Jessup M, Cappola TP. Multiple biomarkers for risk prediction in chronic heart failure. Circ Heart Fail. 2012;5:183–90. 22361079 10.1161/CIRCHEARTFAILURE.111.965020 PMC3387487

[38] Lupón J, Gaggin HK, de Antonio M, Biomarker-assist score for reverse remodeling prediction in heart failure: The ST2-R2 score. Int J Cardiol. 2015;184:337–43. 25734941 10.1016/j.ijcard.2015.02.019

[39] Tayal U, Prasad SK. Myocardial remodelling and recovery in dilated cardiomyopathy. JRSM Cardiovasc Dis. 2017;6:2048004017734476. 10.1177/2048004017734476 PMC5637962 29051817

[40] Sun Y, Chen X, Zhang Y, Yu Y, Zhang X, Si J, Ding Z, Xia Y, Tse G, Liu Y. Reverse Atrial Remodeling in Heart Failure With Recovered Ejection Fraction. J Am Heart Assoc. 2023;12: e026891. 10.1161/JAHA.122.026891 PMC9939067 36645090

[41] Swat SA, Cohen D, Shah SJ, Baseline longitudinal strain predicts recovery of left ventricular ejection fraction in hospitalized patients with nonischemic cardiomyopathy. J Am Heart Assoc. 2018;7:e09841. 10.1161/JAHA.118.009841 PMC6474980 30371257

[42] Adamo L, Perry A, Novak E, Makan M, Lindman BR, Mann DL. Abnormal global longitudinal strain predicts future deterioration of left ventricular function in heart failure patients with a recovered left ventricular ejection fraction. Circ Heart Fail. 2017;10:e003788. 10.1161/CIRCHEARTFAILURE.116.003788 PMC5505492 28559418

[43] Gulati A, Jabbour A, Ismail TF, Association of fibrosis with mortality and sudden cardiac death in patients with nonischemic dilated cardiomyopathy. JAMA. 2013;309:896–908. 23462786 10.1001/jama.2013.1363

[44] Masci PG, Schuurman R, Andrea B, Myocardial fibrosis as a key determinant of left ventricular remodeling in idiopathic dilated cardiomyopathy. Circ Cardiovasc Imaging. 2013;6:790–9. 23934992 10.1161/CIRCIMAGING.113.000438

[45] Kida K, Yoneyama K, Kobayashi Y, Takano M, Akashi YJ, Miyake F. Late gadolinium enhancement on cardiac magnetic resonance images predicts reverse remodeling in patients with nonischemic cardiomyopathy treated with carvedilol. Int J Cardiol. 2013;168:1588–9. 23416019 10.1016/j.ijcard.2013.01.043

[46] Puntmann VO, Carr-White G, Jabbour A, T1-Mapping and outcome in nonischemic cardiomyopathy. JACC Cardiovasc Imaging. 2016;9:40–50. 26762873 10.1016/j.jcmg.2015.12.001

[47] Liu D, Wang M, Murthy V, Myocardial recovery in recent onset dilated cardiomyopathy: role of CDCP1 and cardiac fibrosis. Circ Res. 2023;133:810–25. 37800334 10.1161/CIRCRESAHA.123.323200 PMC10746262

[48] Hazebroek MR, Moors S, Dennert R, Prognostic relevance of gene-environment interactions in patients with dilated cardiomyopathy. J Am Coll Cardiol. 2015;66:1313–23. 26383716 10.1016/j.jacc.2015.07.023

[49] Herman DS, Lam L, Taylor MRG, Truncations of titin causing dilated cardiomyopathy. N Engl J Med. 2012;366:619–28. 22335739 10.1056/NEJMoa1110186 PMC3660031

[50] Ware JS, Li J, Mazaika E, Shared genetic predisposition in peripartum and dilated cardiomyopathies. N Engl J Med. 2016;374:233–41. 26735901 10.1056/NEJMoa1505517 PMC4797319

[51] Jansweijer JA, Nieuwhof K, Russo F, Truncating titin mutations are associated with a mild and treatable form of dilated cardiomyopathy. Eur J Heart Fail. 2017;19:512–21. 27813223 10.1002/ejhf.673

[52] Mann DL, Bristow MR. Mechanisms and Models in Heart Failure. Circulation. 2005;111:2837–49. 15927992 10.1161/CIRCULATIONAHA.104.500546

[53] Tseliou E, Lavine KJ, Wever-Pinzon O, Topkara VK, Meyns B, Adachi I, Zimpfer D, Birks EJ, Burkhoff D, Drakos SG. Biology of myocardial recovery in advanced heart failure with long-term mechanical support. J Heart Lung Transplant. 2022;41:1309–23. 35965183 10.1016/j.healun.2022.07.007

[54] Dandel M, Hetzer R. Recovery of failing hearts by mechanical unloading: Pathophysiologic insights and clinical relevance. Am Heart J. 2018;206:30–50. 30300847 10.1016/j.ahj.2018.09.004

[55] Boulet J, Mehra MR. Left ventricular reverse remodeling in heart failure: remission to recovery. Struct Heart. 2021;5:466–81.

[56] Drakos SG, Badolia R, Makaju A, Distinct transcriptomic and proteomic profile specifies patients who have heart failure with potential of myocardial recovery on mechanical unloading and circulatory support. Circulation. 2023;147:409–24. 36448446 10.1161/CIRCULATIONAHA.121.056600 PMC10062458

[57] Amrute JM, Lai L, Ma P, Defining cardiac functional recovery in end-stage heart failure at single-cell resolution. Nat Cardiovasc Res. 2023;2:399–416. 37583573 10.1038/s44161-023-00260-8 PMC10426763

[58] Margulies KB, Matiwala S, Cornejo C, Olsen H, Craven WA, Bednarik D. Mixed Messages. Circ Res. 2005;96:592–9. 15718504 10.1161/01.RES.0000159390.03503.c3

[59] Weinheimer CJ, Kovacs A, Evans S, Matkovich SJ, Barger PM, Mann DL. Load-dependent changes in left ventricular structure and function in a patho-physiologically relevant murine model of reversible heart failure. Circ Heart Fail. 2018;11:e004351. 10.1161/CIRCHEARTFAILURE.117.004351 PMC5935139 29716898

[60] Hammer Y, Yosef M, Khalatbari S, Aaronson KD. Heart failure with recovered ejection fraction in patients with nonischemic cardiomyopathy: characteristics, outcomes, and long-term follow-up. J Cardiac Fail. 2023;29:1593–602. 10.1016/j.cardfail.2023.06.022 37451602

[61] Merlo M, Stolfo D, Anzini M, Negri F, Pinamonti B, Barbati G, Ramani F, Di Lenarda A, Sinagra G. Persistent recovery of normal left ventricular function and dimension in idiopathic dilated cardiomyopathy during long-term follow-up: does real healing exist? J Am Heart Assoc. 2015;4(1):e001504. 10.1161/JAHA.114.001504 PMC4330074 25587018

[62] Perry AS, Mudigonda P, Huang GS, Qureshi B, Cheng RK, Levy WC, Li S. Long-term outcomes and risk stratification of patients with heart failure with recovered ejection fraction. Am J Cardiol. 2022;173:80–7. 35382925 10.1016/j.amjcard.2022.03.006

[63] Manca P, Stolfo D, Merlo M, Gregorio C, Cannatà A, Ramani F, Nuzzi V, Lund LH, Savarese G, Sinagra G. Transient versus persistent improved ejection fraction in non-ischaemic dilated cardiomyopathy. Eur J Heart Fail. 2022;24:1171–9. 35460146 10.1002/ejhf.2512

[64] Van Kirk J, Fudim M, Green CL, Karra R. Heterogeneous outcomes of heart failure with better ejection fraction. J Cardiovasc Transl Res. 2020;13:142–50. 31721131 10.1007/s12265-019-09919-9 PMC7170767

[65] McElderry B, O’Neill T, Griffin BP, Kalahasti V, Barzilai B, Brateanu A. Factors associated with maintenance of an improved ejection fraction: an echocardiogram-based registry study. J Am Heart Assoc. 2023;12:e031093. 10.1161/JAHA.123.031093 PMC10727417 37889194

[66] Janwanishstaporn S, Cho JY, Feng S, Brann A, Seo JS, Narezkina A, Greenberg B. Prognostic value of global longitudinal strain in patients with heart failure with improved ejection fraction. JACC: Heart Failure. 2022;10(1):27–37. 34969494 10.1016/j.jchf.2021.08.007

[67] Devgun JK, Kennedy S, Slivnick J, Garrett Z, Dodd K, Derbala MH, Ortiz C, Smith SA. Heart failure with recovered ejection fraction and the utility of defibrillator therapy: a review. ESC Heart Failure. 2022;9:1–10. 34953039 10.1002/ehf2.13729 PMC8787956

[68] Park J-S, Kim J-W, Seo K-W, Choi B-J, Choi S-Y, Yoon M-H, Hwang G-S, Tahk S-J, Shin J-H. Recurrence of left ventricular dysfunction in patients with restored idiopathic dilated cardiomyopathy. Clin Cardiol. 2014;37:222–6. 24452755 10.1002/clc.22243 PMC6649465

[69] Takada T, Matsuura K, Minami Y, Abe T, Yoshida A, Kishihara M, Watanabe S, Shirotani S, Jujo K, Hagiwara N. Prognosis and diastolic dysfunction predictors in patients with heart failure and recovered ejection fraction. Sci Rep. 2022;12:8768. 35610337 10.1038/s41598-022-12823-z PMC9130289

[70] He Y, Ling Y, Guo W, Prevalence and prognosis of HFimpEF developed from patients with heart failure with reduced ejection fraction: systematic review and meta-analysis. Front Cardiovasc Med. 2021;8:757596. 34901217 10.3389/fcvm.2021.757596 PMC8655693

